# Genomic insights into selected multidrug-resistant and ESBL-producing uropathogenic *Escherichia coli* isolates in central inner Mongolia

**DOI:** 10.3389/fmicb.2026.1844989

**Published:** 2026-05-29

**Authors:** Jingru Zhang, Shengyuan Wen, Bingzheng Duan, Xuefen Jing, Kai Yang, Xiaomeng Zhai, Ziling Liu

**Affiliations:** 1 The Clinical Laboratory of Baotou Central Hospital, Baotou, China; 2 Baotou Medicine College, Inner Mongolia University of Science and Technology, Baotou, China; 3 Department of Laboratory Medicine, the First Affiliated Hospital of Baotou Medical College, Inner Mongolia University of Science and Technology, Baotou, China

**Keywords:** *Escherichia coli*, extended-spectrumβ-lactamase (ESBL), MDR, phylogenetic group, UPEC, virulence factors, whole-genome sequencing

## Abstract

**Objective:**

The emergence of multidrug-resistant (MDR) and extended-spectrum β-lactamase (ESBL)-producing UPEC has become a global therapeutic challenge. This study aimed to characterize the genomic characteristics, antimicrobial resistance mechanisms, and virulence determinants of selected MDR and ESBL-producing UPEC isolates from central Inner Mongolia.

**Methods:**

A total of 78 *Escherichia coli* isolates were collected from patients with urinary tract infections (UTIs) at Baotou Central Hospital between January 2023 and January 2024. Among them, 28 MDR and ESBL-positive isolates were subjected to whole-genome sequencing using the Illumina platform. Bioinformatics analyses were performed to determine phylogroups, sequence types (STs), antimicrobial resistance genes (ARGs), virulence genes, and plasmid replicons. Statistical analysis was conducted using SPSS 26.0.

**Results:**

The dominant phylogroups were B2 (35.7%) and D (32.1%). The major STs included ST131 (25.0%), ST38 (14.2%), ST69 (10.7%), and ST1193 (10.7%). All isolates harbored *blaCTX-M* genes and *gyrA* S83L mutations. A total of 47 ARGs were identified, among which *mph(A)* (56.6%), *aadA5* (50.0%), and *sul1* (50.0%) were the most prevalent. Virulence genes *fimH, csgA*, and *traT* were widely distributed. Seven strains belonging to ST131 and ST1193 carried a broad repertoire of virulence-associated genes. *IncF* plasmids were detected in 85.7% of isolates and were strongly associated with MDR gene clusters. Additionally, *traT* was identified on *IncB/O/K/Z* plasmids in five isolates.

**Conclusion:**

Within the MDR and ESBL-producing UPEC subpopulation in this region, high-risk clones ST131 and ST1193 are predominant. The co-occurrence of resistance and virulence genes on common plasmid types (e.g., IncF) suggests a potential for co-dissemination, which may contribute to the spread of these high-risk clones.

## 1 Introduction

Urinary tract infections (UTIs) represent one of the most prevalent infectious diseases worldwide. The high incidence of UTIs has imposed a substantial clinical and economic burden on healthcare systems and public health (Salh, 2022). Uropathogenic *Escherichia coli* (UPEC) is the primary etiological agent of UTIs and can colonize and invade urinary tract tissues (Whelan et al., 2024). The enhancement of its pathogenicity depends on the co-expression of virulence genes, which can be horizontally transferred via mobile genetic elements (MGEs) including integrons, transposons, and plasmids (Morales et al., 2023). UPEC harbors a diverse array of virulence factors with specific roles: adhesins such as *fimH* and *csgA* facilitate the initial adhesion of bacteria to urinary tract epithelial cells (Hashimoto et al., 2017), cytotoxins such as *hlyE* cause host tissue injury (Raeispour and Ranjbar, 2018), iron uptake systems (e.g., *iutA* and *fyuA*) promote bacterial survival and growth in the iron-limited host environment (Gebremedhin et al., 2025), and immune evasion factors such as *kpsMII* and *ompT* enable pathogens to evade host immune defenses. Collectively, these virulence attributes enable UPEC to efficiently colonize, invade, and persist during UTIs (Zagaglia et al., 2022). Notably, the carriage of key virulence factors is closely associated with the phylogenetic group of the strain. Multilocus Sequence Typing (MLST) classifies *E. coli* into seven major phylogenetic groups: A, B1, B2, C, D, E, and F (Beghain et al., 2018). Marked differences exist in the virulence gene profiles and ecological adaptability among different phylogenetic group. Groups A and B1 mainly comprise low-virulence commensal strains (Kiiru et al., 2025), whereas Groups B2 and D are strongly associated with highly pathogenic extraintestinal isolates, including UPEC (Hyun et al., 2021). The disparity in pathogenic potential arises from intrinsic variations in the genetic evolution of bacterial strains and selective pressures driving horizontal gene transfer. Among MGEs, plasmids act as critical vectors for the horizontal dissemination of virulence and antimicrobial resistance genes (ARGs) (Pedersen et al., 2020), facilitating the rapid evolution and dissemination of pathogenic and multidrug-resistant lineages (Zhang et al., 2023). This mechanism not only promotes the transmission of ARGs (e.g., β-lactamases) across phylogenetic groups but also facilitates the convergence of high virulence and multidrug resistance in pathogenic strains (Alves et al., 2018; Parras-Moltó et al., 2025). Therefore, this study aims to characterize the molecular epidemiology, virulence, and resistance profiles of UPEC in this region using whole-genome sequencing, in order to provide a scientific basis for the optimization of local clinical treatment strategies and infection control measures.

## 2 Methods

### 2.1 Bacterial strains and identification

Midstream urine specimens were collected from inpatients and outpatients with suspected urinary tract infections (UTIs) at Baotou Central Hospital between January 2023 and January 2024. The urine specimens were cultured on sheep blood agar plates at 37 °C for 18–24 h. Specimens with a bacterial colony count of ≥10∧5 CFU/mL after incubation were selected for further analysis. Escherichia coli (*E. coli*) isolates were identified using the BD Phoenix™ 100 automated microbiology system. The following exclusion criteria were applied: duplicate isolates from the same patient (only the first isolate per patient was retained), contaminated specimens (urine samples yielding three or more bacterial species), and polymicrobial infections (samples with more than one significant bacterial species). After applying these criteria, a total of 78 non-duplicate *E. coli* isolates were collected, representing monomicrobial UTIs. Among these, 28 multidrug-resistant (MDR) and ESBL-positive isolates were selected as the study cohort for whole-genome sequencing. MDR was defined as resistance to at least three distinct classes of antimicrobial agents (e.g., β-lactams, fluoroquinolones, aminoglycosides), and ESBL production was confirmed by the BD Phoenix™ 100 system.

### 2.2 Antimicrobial susceptibility testing

The antimicrobial susceptibility of 28 bacterial strains to 20 antibiotics was determined using the BD Phoenix™ automated system. The tested antibiotics included: carbapenems (imipenem, meropenem); cephalosporins (cefazolin, first-generation; ceftazidime and cefotaxime, third-generation; cefepime, fourth-generation); monobactam (aztreonam); penicillins (ampicillin, piperacillin); penicillin/β-lactamase inhibitor combinations (amoxicillin-clavulanic acid, ampicillin-sulbactam, piperacillin-tazobactam); aminoglycosides (amikacin, gentamicin); fluoroquinolones (ciprofloxacin, levofloxacin, moxifloxacin); sulfonamide (trimethoprim-sulfamethoxazole); tetracycline (tetracycline); and phenicol (chloramphenicol). Interpretation of susceptibility results was performed in accordance with the clinical and laboratory standards published by the Clinical and Laboratory Standards Institute (CLSI) (Wayne, 2022), with *Escherichia coli* ATCC 25922 used as the quality control strain.

### 2.3 Whole-genome sequencing and bioinformatics analysis

Genomic DNA was extracted from all 28 selected MDR and ESBL-positive UPEC isolates and randomly fragmented using a Covaris ultrasonicator. Library construction was performed via end repair, A-tailing, adapter ligation, and PCR amplification. Whole-genome sequencing was conducted on the Illumina platform with paired-end 150 bp (PE150) reads. Raw sequencing reads were quality-filtered using fastp (v0.20.0; https://github.com/OpenGene/fastp) to obtain clean data.

MLST was performed using MLST 2.0.4 according to the seven-gene scheme (*adk, fumC, gyrB, icd, mdh, purA, recA*). Phylogenetic groups were determined using the ClermonTyping web server (http://clermontyping.iame-research.center/). ARGs and virulence factors were identified using ResFinder 3.2 and VirulenceFinder 2.0, respectively. The thresholds were set at 90% identity and 60% coverage for ARGs, and 85% identity and 60% coverage for virulence genes. Plasmid replicons were characterized using PlasmidFinder 2.0. Phylogenetic trees were constructed with 1,000 bootstrap replicates, and nodes with bootstrap support ≥70% were considered well-supported.

### 2.4 Definition of high virulence gene score

To semi-quantitatively assess the predicted virulence potential of each isolate, one point was assigned for the presence of each of the following categories of virulence-associated genes: adhesins (e.g., *fimH, papC, papA*), toxins (e.g., *hlyA, sat, hlyE)*, iron uptake systems (e.g., iutA, fyuA, ChuA), protectins/serum resistance (e.g., *kpsMII, traT, ompT*), and miscellaneous virulence factors (e.g., usp, iha, pic). A total score was calculated for each isolate. Isolates with a virulence gene count above the cohort median were classified as the “high virulence gene load” group.

### 2.5 Statistical analysis

All statistical analyses were performed using SPSS 26.0 (IBM Corp., Armonk, NY, USA) software. Fisher's exact test was used to evaluate the associations between different phylogenetic/subgroup systems and the distribution of virulence genes in *Escherichia coli*. A *P-*value < 0.05 was considered statistically significant. Spearman correlation analysis was conducted using the *R* language to construct a co-occurrence network diagram of plasmids and ARGs, with a correlation of 0.6 and a significance of 0.05.

## 3 Results

### 3.1 Analysis of antibiotic susceptibility test results

**Figure 1:**
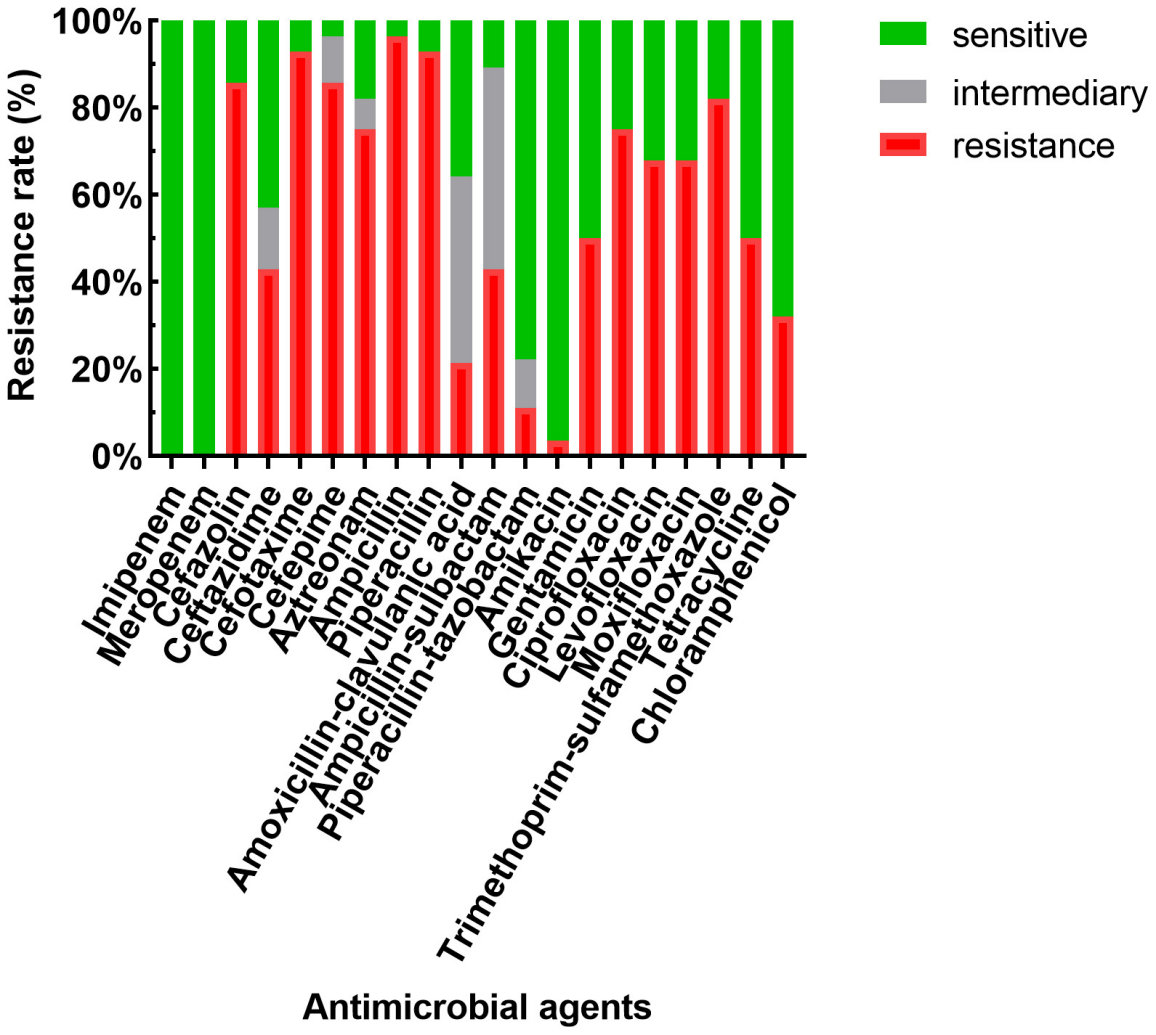
Distribution of antimicrobial resistance among 28 selected MDR and ESBL-producing UPEC isolates.

All 28 selected MDR and ESBL-positive UPEC clinical isolates were resistant to at least three classes of antibiotics, indicating a severe multidrug-resistant phenotype. Antibiotics with resistance rates ≥90% included ampicillin, piperacillin, cefepime, cefazolin, and cefotaxime. The resistance rates for sulfamethoxazole, ciprofloxacin, levofloxacin, and moxifloxacin ranged from 70 to 90%. The resistance rates for ceftazidime, ampicillin/sulbactam, gentamicin, and chloramphenicol ranged from 40 to 60%. The resistance rates for piperacillin-tazobactam and amikacin were relatively low, at 14.2% for both. All strains were susceptible to carbapenems (imipenem and meropenem), as shown in Figure 1.

### 3.2 Genomic features

Genome sizes ranged from 4.92 to 5.56 Mb, with G+C content ranging from 51.17 to 51.64%. The number of predicted coding sequences varied from 4,703 to 5,372. Additional genomic features, including tRNA/rRNA counts, genomic islands, and MGEs, are summarized in Table 1.

**Table 1:** Genomic characteristics of the 28 selected MDR and ESBL-positive UPEC isolates.

Range	Characteristic
Genome size/bp	4,918,112–5,563,703
Number of predicted coding genes	4,703–5,372
G+C content/%	51.17–51.64
Number of tRNAs	77–92
Number of 16rRNAs	1–9
Number of sRNAs	102–181
Number of CRISPRs	5–38
Number of transposons	31–42
Number of dispersed repeat sequences	14–45
Number of genomic islands	7–17
Total length of genomic islands/bp	7,5673–225,813
Number of T3SS effector proteins	169–207
Number of secreted proteins	303–337
Number of transmembrane proteins	1,102–1,236
Number of signal peptides	378–424

^*^T3SS: Type III secretion system, The input amino acid sequence is predicted using the software effectivet3.

### 3.3 Molecular subtyping characteristics

**Figure 2:**
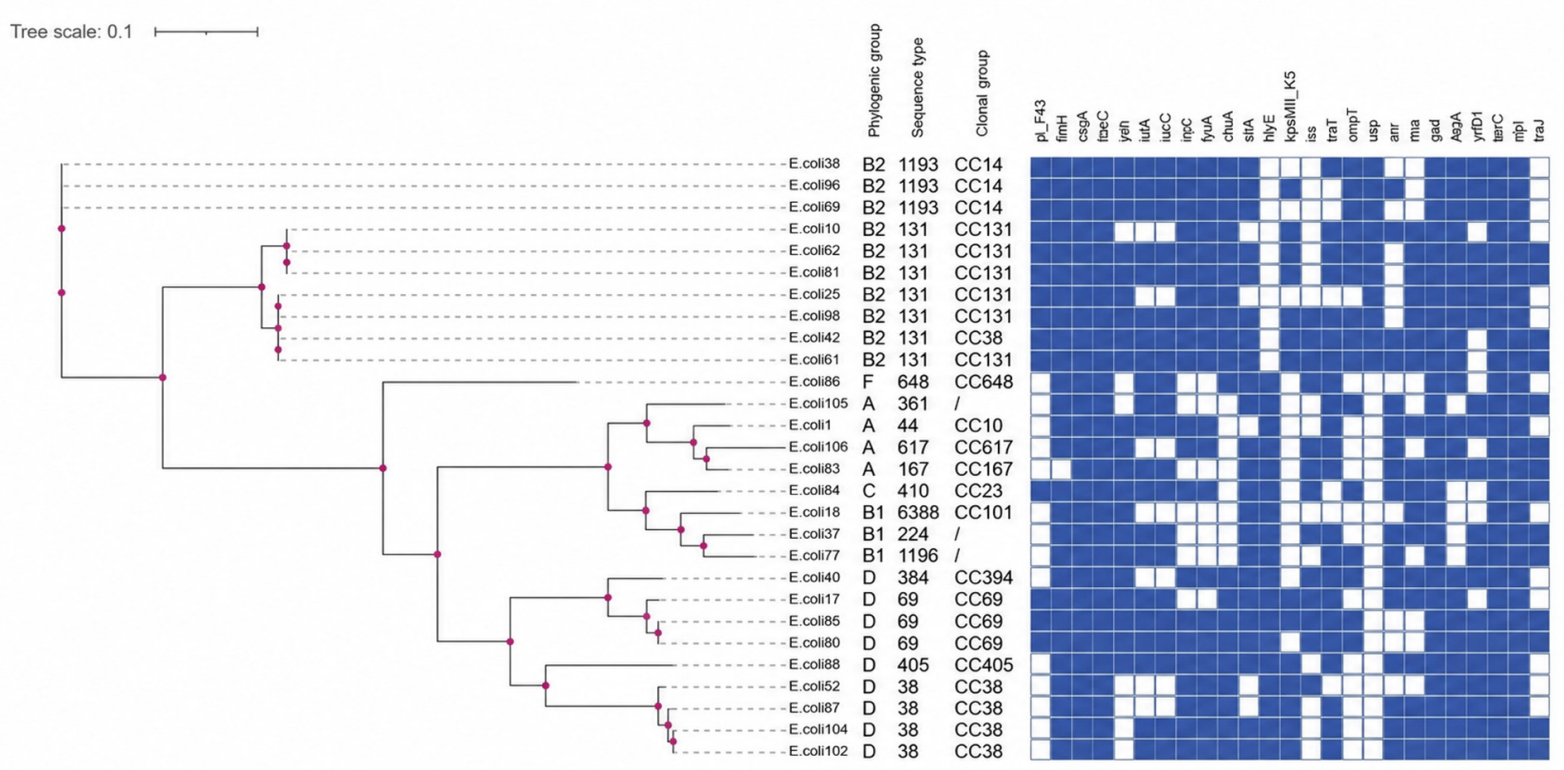
Phylogenetic tree map of 28 UPEC strains. *Bootstrap values (1,000 replicates) are shown at the nodes; only values ≥70% are displayed. The scale bar (0.1) indicates the number of nucleotide substitutions per site. ST, sequence type; CC, clonal complex; *E. coil, Escherichia coli*.

The phylogenetic typing revealed that among the 28 selected MDR and ESBL-positive UPEC isolates, Group B2 (10/28, 35.7%) and Group D (9/28, 32.1%) were the dominant phylogenetic groups, while the remaining strains were categorized as Group A (4/28, 14.2%), Group B1 (3/28, 10.7%), Group C (1/28, 3.5%), and Group F (1/28, 3.5%). MLST identified 15 distinct sequence types (STs). ST131 (7/28, 25.0%) was the most prevalent ST, followed by ST38 (4/28, 14.2%), ST69 and ST1193 (3/28, 10.7% each). As shown in Figure 2, core gene phylogenetic analysis demonstrated that Group D strains formed a distinct and well-supported major clade, which was further divided into two primary subclades corresponding to ST38 and ST69, accounting for 78% of this clade. Group B2 formed two separate and highly robust subclades that were consistent with the two dominant clones, ST1193 and ST131, respectively. The reliability of these major clades was supported by bootstrap analysis with 1,000 replicates (bootstrap values > 70%).

### 3.4 Analysis results of virulence factors

Among the 28 selected MDR and ESBL-positive UPEC isolates, a large number of virulence genes associated with adhesion, invasion, iron acquisition, secretion systems, and toxin synthesis was identified. *E. coli s*train 87 harbored the highest number of virulence genes, fourteen strains (80, 17, 81, 87, 42, 61, 85, 86, 98, 38, 69, 96, 102, 105) harbored a larger number of virulence-associated genes compared to the other isolates. Using the cohort median (23.5) as the threshold, 14 isolates were classified as the high virulence gene load group. Among these, phylogroup B2 accounted for 50.0% (7/14) and phylogroup D for 35.7% (5/14), together comprising 85.7% of the high–virulence group. This indicates a strong association between high virulence potential and phylogroups B2 and D, with B2 being the most represented. The virulence factors *fimH* (100%), *fdeC* (100%), *gad* (100%), *nlpI* (100%), and *terC* (100%) were detected in all UPEC isolates. As shown in Table 2: *papC, fyuA/irp2, aslA, usp*, and c*huA* were predominantly distributed in phylogenetic group B2, whereas *fimH, csgA, terC, nlpI*, and *traT* were widely distributed across all groups. The virulence factors *hlyE, kpsMII_K5*, e*ilA*, and *air* were mainly present in Group D. Fisher's exact test demonstrated that the distribution of 14 virulence-associated genes varied significantly among the phylogenetic groups (*P* < 0.05). Phylogroups B2 and D were associated with the carriage of the majority of these genes.

**Table 2:** Relationship between virulence genes and phylogenetic clades.

Gene	A (*n* = 4)	B1 (*n* = 3)	B2 (*n* = 10)	D (*n* = 9)	Total	*P*
*fimH*	3	3	10	9	25	>0.05
*papC*	0	1	5	1	7	>0.05
*sitA*	3	3	8	7	21	>0.05
*iucC*	3	2	8	6	19	>0.05
*anr*	4	2	4	6	16	>0.05
*hlyE*	4	3	0	9	16	<0.05
*csgA*	4	3	10	9	26	–
*yehA*	3	3	9	5	20	>0.05
*fyuA*	2	0	10	8	20	<0.05
*irp2*	2	0	10	8	20	<0.05
*gad*	4	3	10	9	26	–
*yghJ*	3	2	7	8	20	>0.05
*terC*	4	3	10	9	26	–
*nlpI*	4	3	10	9	26	–
*hha*	2	2	7	6	17	>0.05
*aslA*	3	0	10	9	22	<0.05
*ompT*	1	1	9	3	14	<0.05
*kpsMII_K*5	0	0	7	7	14	<0.05
*kpsE*	0	0	3	3	6	>0.05
*chuA*	0	0	10	9	19	<0.05
*papA_F43*	0	0	10	3	13	<0.05
*usp*	0	0	10	0	10	<0.05
*iha*	0	0	8	2	10	<0.05
*sat*	0	0	8	1	9	<0.05
*lpfA*	0	3	0	4	7	<0.05
*eilA*	0	0	0	8	8	<0.05
*air*	0	0	0	9	9	<0.05
*aamR*	0	0	1	5	6	>0.05
*traT*	4	2	7	8	21	>0.05

^*^Phylogroups C (*n* = 1) and F (*n* = 1) were excluded from statistical analysis due to small sample sizes and are presented only descriptively in the main text. *P* < 0.05 was considered statistically significant. “–” indicates that the *P*-value could not be calculated due to identical distribution across all groups (100% or 0%).

### 3.5 Analysis of ARGs profile

A total of 47 ARGs belonging to nine major categories were identified by whole-genome sequencing. All strains carried a considerable number of ARGs, ranging from six and 17 per strain, with a complex ARGs composition. Among the resistance gene categories, β-lactam resistance genes showed the highest prevalence (89.3%), followed by aminoglycosides (72.1%), sulfonamides (72.1%), trimethoprims (71.4%), and macrolides (67.8%). Aminoglycoside resistance genes displayed the greatest diversity, with 17 distinct genes identified in total. The gene *mph(A)* was detected in 56.6% (17/28) of the isolates, followed by *aadA5* (50.0%, 15/28), *sul1* (50.0%, 15/28), *dfrA17* (43.3%, 13/28), *tet(A)* (43.3%, 13/28), and *blaTEM-1B* (33.3%, 10/28), indicating that these genes were highly prevalent.

### 3.6 Analysis of ARGs associated with plasmid replicons and MGEs

**Figure 3:**
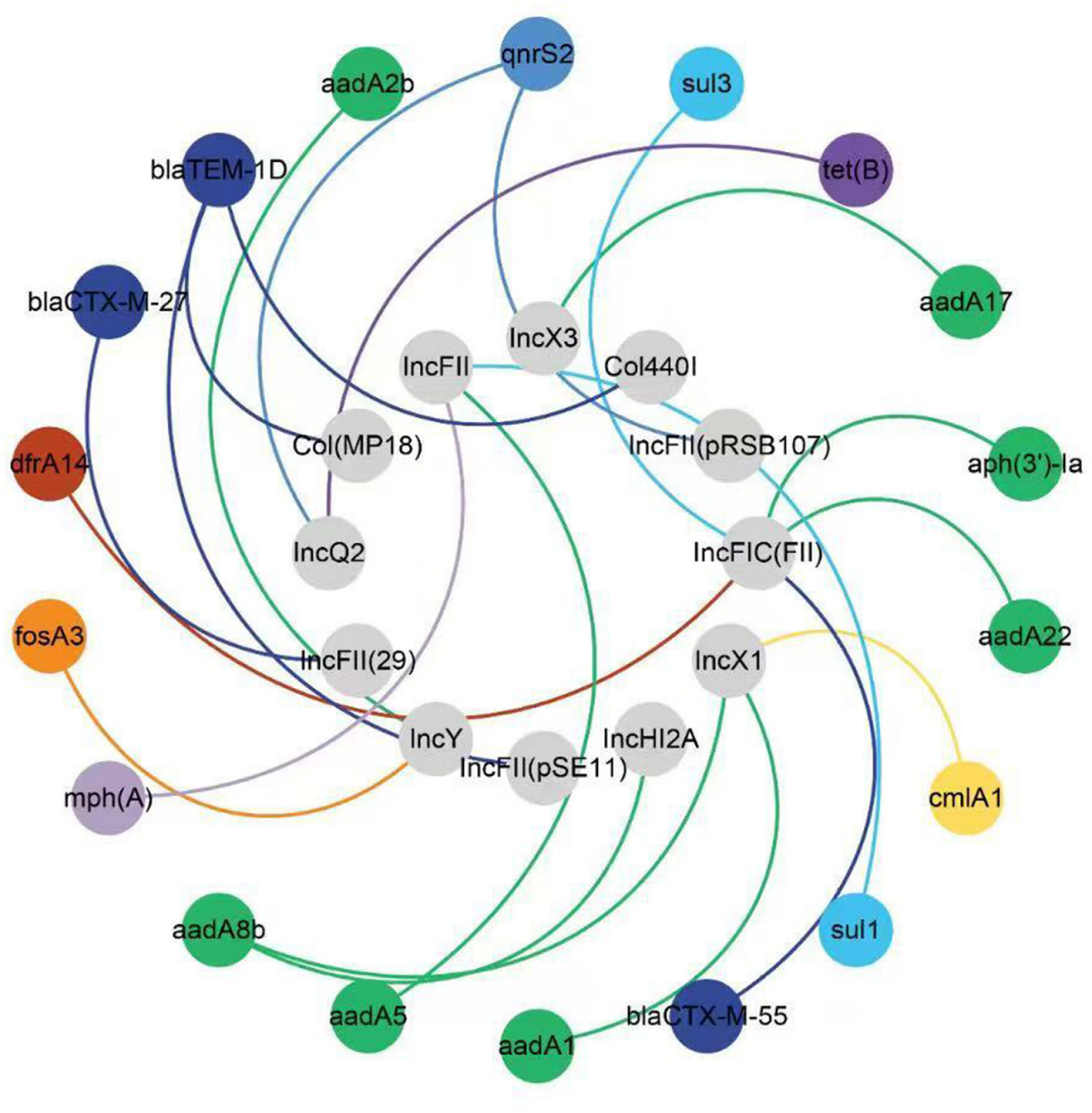
Co-occurrence network of plasmid replicons and ARGs. *Nodes represent plasmid replicon types (**blue**) and ARGs (**red**). Edges indicate significant positive correlations (*P* < 0.05).

The plasmid replicon typing of 28 isolates identified 31 different replicon types, indicating a diverse but structurally conserved plasmid repertoire. Replicons of the *IncF* incompatibility group were predominant; the *IncFIB* (AP001918) subtype showed the highest prevalence (67.8%), followed by *IncFIA* (50%) and *IncFII* (39.2%). Correlation analysis between replicon types and acquired resistance genes revealed specific associations (Figure 3). It is worth noting that the coexistence of *IncFII* with mph (A)*, sul1, and aadA5* genes shows a strong correlation. In addition, *IncFIC* is significantly associated with ARGs clusters containing *sul3, blaCTX-M-55, aadA22*, and *aph (3')-la*. The extensive IncF plasmid population is associated with the majority of detected ARGs, suggesting a potential role for IncF plasmids in the dissemination of MDR phenotypes in this collection.

### 3.7 Analysis of class 1 integrons

Whole-genome sequencing analysis revealed the presence of complete class one integrons in 15 of the 28 (53.6%) MDR/ESBL-producing UPEC isolates. The most frequently identified gene cassette array was aadA5-dfrA17-qacE-sul1 (found in 11 isolates), conferring resistance to aminoglycosides, trimethoprim, sulfonamides, and disinfectants. Promoter analysis: for isolate H62, the Pc promoter was identified as a strong promoter (PcS). No weak promoters (PcW) were detected in any isolate.

## 4 Discussion

This work used comparative genomics techniques to examine 28 isolates of *E. coli* across three dimensions: genomic characteristics, molecular attributes, and evolutionary genetic linkages, encompassing their phylogenetic classifications, virulence genes, and ARGs. The results indicated substantial variations in several markers, including genome size and predicted coding gene count, across the 28 isolates of *E. coli*. Genomic diversity can result in distinct differences in genome stability, biological traits, pathogenicity, and adaptation of strains, offering a potential biological foundation for the survival and reproduction of UPEC in various environments and host organisms.

This study identified the predominant phylogenetic groups as B2 (35.7%) and D (32.1%), consistent with the findings reported by researchers from neighboring Mongolia (Munkhdelger et al., 2017). The combined proportion of groups A and B1 accounted for 10.7% of isolates, which is higher than that reported in other studies (Lee et al., 2016). The B2 cluster included of the two high-risk clones ST1193 and ST131, while the D cluster mainly comprised ST69 and ST38. These four STs are precisely the most significant clones that have led to the global prevalence of MDR parenteral infections. As different lineages carry distinct virulence gene profiles, these bacteria exhibit uropathogenicity (Forsyth et al., 2018). All isolates harbored *csgA, fdeC, gad, terC, and nlpI* genes, which are essential virulence factors supporting survival and pathogenesis of UPEC in the urinary tract (Terlizzi et al., 2017). The virulence genes *papC, fyuA/irp2, aslA, usp*, and *chuA* were significantly concentrated in the B2 group, consistent with the high-virulence characteristic of the B2 strain. Notably, *fyuA/irp2* (yersiniabactin-related genes) and *usp* (urinary system specific protein) served as nearly exclusive markers for Group B2 (Whelan et al., 2024). Group D also represented an important reservoir of virulence genes, with *hlyE, kpsMII_K5, eilA and air* significantly enriched in this group. Of note, *hlyE* (hemolysin E) was absent in group B2 but unique to group D, whereas *eilA* and *air* were detected only in group D. These findings indicate marked divergence in virulence gene profiles among phylogenetic groups. Several “broad-spectrum” virulence genes, including *fimH, csgA, terC, nlpI*, and *traT* were widely distributed across all groups but showed clear preferential distribution, being predominantly present in groups B2 and D and absent or less frequent in groups C and F. Clinically, isolates belonging to phylogenetic group B2 or D should raise high alert due to their association with major drug-resistant clones, to guide empirical antimicrobial therapy and infection control. Based on their virulence gene profiles, fourteen isolates were identified as carrying an extensive set of virulence-associated genes. Its molecular characteristics reveal a particularly severe clinical challenge: the dominant clones ST131 and ST1193 combine high virulence and extensive multidrug resistance, in agreement with the previous observations by Francesca Caballero et al. (Caballero et al., 2025).

All isolates carried the *blaCTX-M* gene, consistent with the high rate of cefotaxime resistance (92.8%), indicating that third-generation cephalosporins are not suitable for empirical therapy of such infection. More notably, the quinolone resistance mechanism was observed: all isolates carried the *gyrA* S83L mutation, with two-thirds harboring the S83L+D87N double mutation, while plasmid-mediated *qnr* protective genes were exceedingly rare. Under strong selective pressure from fluoroquinolones, chromosomal target site mutations have become the dominant evolutionary pathway for ST131 clones to acquire and maintain high-level quinolone resistance (Basu and Mukherjee, 2019). The high prevalence of the gyrA S83L mutation, even in fluoroquinolone-susceptible isolates, suggests that this mutation may not impose a significant fitness cost. Otherwise, in the absence of antibiotic selection pressure, it would likely be eliminated from the population. It has been hypothesized that specific gyrA mutations, including S83L, may confer a fitness advantage to certain *E. coli* clones beyond their direct role in quinolone resistance (Boueroy et al., 2023; Fuzi and Sokurenko, 2023). However, distinguishing between the absence of fitness cost and an actual fitness benefit would require direct experimental validation.

This study identified diverse plasmid replicon types in UPEC isolates, with most isolates harboring multiple plasmids concurrently. Among them, IncFIA and IncFIB replicons were the most prevalent and stably maintained (Ravi et al., 2018). IncF plasmids showed significant associations with MDR genes, including the macrolide resistance gene *Mph(A)*, aminoglycoside resistance gene *AadA5*, sulfonamide resistance gene S*ul*, trimethoprim resistance gene *dfrA*, tetracycline resistance gene *Tet(A)*, and β*-lactamase* gene *blaCTX*. Notably, these genes corresponded to the most prevalent resistance determinants detected. These findings suggest an association between IncF-type plasmids and the co-carriage of MDR and virulence genes, implying that they may serve as potential vectors for their co-dissemination (Irrgang et al., 2017; Pitout and Chen, 2023). Experimental evidence from Iran further supports this mechanism, where conjugation assays confirmed the horizontal transferability of carbapenem resistance via plasmids in UPEC (Zangane Matin et al., 2021).

Plasmid-mediated horizontal gene transfer is the fundamental mechanism underlying multidrug resistance and the co-transmission of high virulence in UPEC. This study demonstrated that the majority of IncF plasmid strains possessed both *bla*CTX-M-15 (resistance) and *TraT* (virulence) genes, establishing a genetic basis for multidrug resistance and transmission advantage. Besides IncF plasmids, we identified IncA/C and *IncY* plasmids, which possess a broad host range and facilitate multidrug resistance and the transfer of virulence-associated genes between strains. The presence of numerous plasmids considerably enhances UPEC's capacity to acquire ARGs via horizontal gene transfer, hence expediting the dissemination of resistance among pathogen populations (Wang et al., 2024). In addition to plasmids, class one integrons were detected in over half (53.6%) of our isolates. Notably, the absence of weak-promoter integrons (PcW) suggests that these clinical isolates are optimized for high-level resistance expression under antibiotic selection pressure.

Notably, in five *E. coli* isolates, the presence of the traT gene was observed in strains also carrying IncB/O/K/Z-type plasmids. While IncF plasmids are traditionally considered primary vectors for traT (Ashraf et al., 2025), our data raise the hypothesis that IncB/O/K/Z plasmids might also be involved in its dissemination (Lefèvre et al., 2023; Campos-Madueno et al., 2023). It is tempting to speculate that a plasmid harboring both resistance and virulence genes could exist on an IncB/O/K/Z backbone, potentially enhancing its clinical risk. However, this hypothesis requires confirmation through long-read sequencing or conjugation experiments (Moglad and Altayb, 2023).

## 5 Conclusion

The comprehensive whole-genome sequencing analysis of 28 selected MDR and ESBL-positive UPEC isolates in central Inner Mongolia demonstrated a high prevalence of MDR and elevated virulence. This investigation indicates that locally circulating UPEC with a high virulence gene load are predominantly group B2 strains, with ST131 as the dominant prevalent clone; additionally, the emerging clone ST1193, which exhibits dissemination potential, was identified. Plasmids appear to play a central role in the co-occurrence of drug resistance and virulence genes in this collection. Our data suggest that the dissemination of traT may not be strictly confined to IncF plasmids, with IncB/O/K/Z-type plasmids representing another potential vehicle. These findings highlight the need for functional studies to elucidate the precise mechanisms of gene transfer and the evolution of these clinically concerning isolates.

## 6 Limitation

This study has several limitations. First, whole-genome sequencing was performed exclusively on 28 multidrug-resistant and ESBL-positive isolates selected from a larger collection of 78 *E. coli* isolates. Therefore, the findings are representative only of this resistant subpopulation and cannot be generalized to all UPEC isolates in Central Inner Mongolia. Second, the sample size of certain phylogenetic groups (e.g., groups C and F, *n* = 1 each) was too small for robust statistical comparisons. Third, virulence potential was assessed based on gene content rather than functional assays. Fourth, clinical data such as indwelling catheter use, prior antibiotic exposure, and recurrence status were not systematically collected and thus could not be correlated with genomic findings. Fifth, whole-genome sequencing was performed using short-read (Illumina) technology, which precludes complete assembly of plasmid structures and definitive identification of co-localization of resistance and virulence genes on the same replicon. Despite these limitations, this study provides valuable genomic insights into the high-risk MDR/ESBL-producing UPEC lineages circulating in this region.

## Data Availability

The whole-genome sequences of 28 *Escherichia coli* strains were submitted to the NCBI GenBank database. The corresponding GenBank accession number is JBWVLN000000000, with the BioSample accession number SAMN56834042 and the NCBI submission ID SUB16096975.

## References

[B1] Alves T. D. S. Lara G. H. B. Maluta R. P. Ribeiro M. G. Leite D. D. S. (2018). Carrier flies of multidrug-resistant *Escherichia coli* as potential dissemination agent in dairy farm environment. Sci. Total Environ. 633, 1345–1351. doi: 10.1016/j.scitotenv.2018.03.304 29758886

[B2] Ashraf Z. Rasool M. H. Aslam B. Ejaz H. Mujahid F. Khurshid M. . (2025). Dynamics of urinary tract infections: a comprehensive study on antimicrobial susceptibility, virulence profiling and molecular epidemiology of uropathogenic *Escherichia coli* from Pakistan. Mol. Biol. Rep. 52:694. doi: 10.1007/s11033-025-10799-3 40637937

[B3] Basu S. Mukherjee M. (2019). Conjugal transfer of PMQR from uropathogenic *E. coli* under high ciprofloxacin selection pressure generates gyrA mutation. Micro. Patho. 132, 26–29. doi: 10.1016/j.micpath.2019.04.021 30999022

[B4] Beghain J. Bridier-Nahmias A. Le Nagard H. Denamur E. Clermont O. (2018). ClermonTyping: an easy-to-use and accurate *in silico* method for Escherichia genus strain phylotyping. Microb. Genom. 4:e000192. doi: 10.1099/mgen.0.000192 29916797 PMC6113867

[B5] Boueroy P. Chopjitt P. Hatrongjit R. Morita M. Sugawara Y. Akeda Y. . (2023). Fluoroquinolone resistance determinants in carbapenem-resistant *Escherichia coli* isolated from urine clinical samples in Thailand. PeerJ 11:e16401. doi: 10.7717/peerj.16401 37953793 PMC10638923

[B6] Caballero F. Martinez-Ventura A. Cuicapuza D. Fajardo-Loyola A. Gutierrez-Ajalcriña R. Soto-Pastrana J. . (2025). Genomic diversity of uropathogenic *Escherichia coli* in clinical isolates from six latin american countries, 2018-2023. Rev. Peruana de Med. Exp. Y Salud Pub. 42, 156–165. doi: 10.17843/rpmesp.2025.422.14299 PMC12377891 40900482

[B7] Campos-Madueno E. I. Aldeia C. Sendi P. Endimiani A. (2023). Escherichia ruysiae may serve as a reservoir of antibiotic resistance genes across multiple settings and regions. Microbiol. Spectr. 11:e0175323. doi: 10.1128/spectrum.01753-23 37318364 PMC10434276

[B8] Forsyth V. S. Armbruster C. E. Smith S. N. Pirani A. Springman A. C. Walters M. S. . (2018). Rapid growth of uropathogenic *Escherichia coli* during human urinary tract infection. mBio 9:e00186. doi: 10.1128/mBio.00186-18 29511075 PMC5844997

[B9] Fuzi M. Sokurenko E. (2023). Commensal fitness advantage may contribute to the global dissemination of multidrug-resistant lineages of bacteria-the case of uropathogenic *E. coli*. Pathogens 12:1150. doi: 10.3390/pathogens12091150 37764958 PMC10536240

[B10] Gebremedhin K. B. Amogne W. Alemayehu H. Bopegamage S. Eguale T. (2025). The role of uropathogenic *Escherichia coli* virulence factors in the development of urinary tract infection. J. Med. Life 18, 701–709. doi: 10.25122/jml-2024-0396 41020073 PMC12467495

[B11] Hashimoto J. Takahashi M. Saito A. Murata M. Kurimura Y. Nishitani C. . (2017). Surfactant protein a inhibits growth and adherence of uropathogenic *Escherichia coli* to protect the bladder from infection. J. Immunol. 198, 2898–2905. doi: 10.4049/jimmunol.1502626 28228557

[B12] Hyun M. Lee J. Y. Kim H. A. (2021). Differences of virulence factors, and antimicrobial susceptibility according to phylogenetic group in uropathogenic *Escherichia coli* strains isolated from Korean patients. Ann. Clin. Microbiol. Antimicrob. 20:77. doi: 10.1186/s12941-021-00481-4 34758824 PMC8579644

[B13] Irrgang A. Falgenhauer L. Fischer J. Ghosh H. Guiral E. Guerra B. . (2017). CTX-M-15-Producing *E. coli* isolates from food products in germany are mainly associated with an IncF-type plasmid and belong to two predominant clonal *E. coli* lineages. Front. Microbiol. 8:2318. doi: 10.3389/fmicb.2017.02318 29209306 PMC5702323

[B14] Kiiru S. Kasiano P. Maina J. Mwaniki J. N. Songoro E. Kariuki S. . (2025). Molecular characterization of multidrug-resistant *E. coli* recovered from diarrheagenic children under 5 years from Mukuru Informal Settlement, Nairobi, Kenya, based on whole-genome sequencing analysis. Microbiol. Spectr. 13:e0142024. doi: 10.1128/spectrum.01420-24 40372033 PMC12131759

[B15] Lee J. H. Subhadra B. Son Y. J. Kim D. H. Park H. S. Kim J. M. . (2016). Phylogenetic group distributions, virulence factors and antimicrobial resistance properties of uropathogenic *Escherichia coli* strains isolated from patients with urinary tract infections in South Korea. Lett. Appl. Microbiol. 62, 84–90. doi: 10.1111/lam.12517 26518617

[B16] Lefèvre S. Njamkepo E. Feldman S. Ruckly C. Carle I. Lejay-Collin M. . (2023). Rapid emergence of extensively drug-resistant Shigella sonnei in France. Nat. Commun. 14:462. doi: 10.1038/s41467-023-36222-8 36709320 PMC9883819

[B17] Moglad E. Altayb H. N. (2023). Genomic characterization of extended spectrum beta lactamases producing multidrug-resistant *Escherichia coli* clinically isolated harboring chromosomally mediated CTX-M-15 from Alkharj, KSA. *Infect. Genet. E*. 116:105526. doi: 10.1016/j.meegid.2023.105526 37977421

[B18] Morales G. Abelson B. Reasoner S. Miller J. Earl A. M. Hadjifrangiskou M. . (2023). The role of mobile genetic elements in virulence factor carriage from symptomatic and asymptomatic cases of *Escherichia coli* bacteriuria. Microbiol. Spectr. 11:e0471022. doi: 10.1128/spectrum.04710-22 37195213 PMC10269530

[B19] Munkhdelger Y. Gunregjav N. Dorjpurev A. Juniichiro N. Sarantuya J. (2017). Detection of virulence genes, phylogenetic group and antibiotic resistance of uropathogenic *Escherichia coli* in Mongolia. J. Infect. Dev. Ctries 11, 51–57. doi: 10.3855/jidc.7903 28141590

[B20] Parras-Moltó M. Lund D. Ebmeyer S. Larsson D. G. J. Johnning A. Kristiansson E. . (2025). The transfer of antibiotic resistance genes between evolutionarily distant bacteria. mSphere 10:e0011425. doi: 10.1128/msphere.00114-25 40459279 PMC12188727

[B21] Pedersen T. Tellevik M. G. Kommedal Ø. Lindemann P. C. Moyo S. J. Janice J. . (2020). Horizontal plasmid transfer among Klebsiella pneumoniae isolates is the key factor for dissemination of extended-spectrum β-Lactamases among Children in Tanzania. mSphere 5:e00428. doi: 10.1128/msphere.00428-20 32669470 PMC7364214

[B22] Pitout J. D. D. Chen L. (2023). The significance of epidemic plasmids in the success of multidrug-resistant drug pandemic extraintestinal pathogenic *Escherichia coli*. Infect. Dis. Ther. 12, 1029–1041. doi: 10.1007/s40121-023-00791-4 36947392 PMC10147871

[B23] Raeispour M. Ranjbar R. (2018). Antibiotic resistance, virulence factors and genotyping of uropathogenic *Escherichia coli* strains. Antimicrob. Resist. Infect. Control 7:118. doi: 10.1186/s13756-018-0411-4 30305891 PMC6171155

[B24] Ravi A. Valdés-Varela L. Gueimonde M. Rudi K. (2018). Transmission and persistence of IncF conjugative plasmids in the gut microbiota of full-term infants. FEMS Microbiol. Ecol. 94:1. doi: 10.1093/femsec/fix158 29161377

[B25] Salh K. K. (2022). Antimicrobial resistance in bacteria causing urinary tract infections. Comb. Chem. High Throughput Screen 25, 1219–1229. doi: 10.2174/1386207324666210622161325 34161207

[B26] Terlizzi M. E. Gribaudo G. Maffei M. E. (2017). UroPathogenic *Escherichia coli* (UPEC) infections: virulence factors, bladder responses, antibiotic, and non-antibiotic antimicrobial strategies. Front. Microbiol. 8:1566. doi: 10.3389/fmicb.2017.01566 28861072 PMC5559502

[B27] Wang X. Zhang H. Yu S. Li D. Gillings M. R. Ren H. . (2024). Inter-plasmid transfer of antibiotic resistance genes accelerates antibiotic resistance in bacterial pathogens. ISME J. 18:32. doi: 10.1093/ismejo/wrad032 PMC10881300 38366209

[B28] Wayne P. (2022). Clinical and Laboratory Standards Institute (CLSI). Performance standards for antimicrobial susceptibility testing, 32nd Edn. Wayne: CLSI.

[B29] Whelan S. Bottacini F. Buttimer C. Finn K. Lucey B. (2024). Whole genome sequencing of uropathogenic *E. coli* from Ireland reveals diverse resistance mechanisms and strong correlation with phenotypic (EUCAST) susceptibility testing. Inf. Gene. Evol. J. Mol. Epidemiol. Evol. Gene. Infect. Dis. 121:105600. doi: 10.1016/j.meegid.2024.105600 38692501

[B30] Zagaglia C. Ammendolia M. G. Maurizi L. Nicoletti M. Longhi C. (2022). Urinary tract infections caused by uropathogenic *Escherichia coli* strains-new strategies for an old pathogen. Microorganisms 10:1425. doi: 10.3390/microorganisms10071425 35889146 PMC9321218

[B31] Zangane Matin F. Rezatofighi S. E. Roayaei Ardakani M. Akhoond M. R. Mahmoodi F. (2021). Virulence characterization and clonal analysis of uropathogenic *Escherichia coli* metallo-beta-lactamase-producing isolates. Ann. Clin. Microbiol. Antimicrob. 20:50. doi: 10.1186/s12941-021-00457-4 34344363 PMC8336094

[B32] Zhang J. Xu Y. Wang M. Li X. Liu Z. Kuang D. . (2023). Mobilizable plasmids drive the spread of antimicrobial resistance genes and virulence genes in Klebsiella pneumoniae. Genom. Med. 15:106. doi: 10.1186/s13073-023-01260-w PMC10691111 38041146

